# Acoustic Control of Spin States in Diamond Nitrogen-Vacancy Centres

**DOI:** 10.1021/acs.nanolett.6c02496

**Published:** 2026-08-12

**Authors:** Emirhan Kutsal, Amir Farokh Payam, Eihab Abdel-Rahman, Mustafa Yavuz, Lijie Li

**Affiliations:** † Department of Electronic and Electrical Engineering, 536010 Swansea University, Swansea SA1 8EN, U.K.; ‡ Nanotechnology and Integrated Bioengineering Centre, School of Engineering, 2596 Ulster University, Belfast BT15 1AP, U.K.; § Waterloo Institute for Nanotechnology (WIN), 504414 University of Waterloo, Waterloo, ON N2L 3G1, Canada; ∥ Department of Systems Design Engineering, University of Waterloo, Waterloo, ON N2L 3G1, Canada; ⊥ Department of Mechanical and Mechatronics Engineering, University of Waterloo, Waterloo, ON N2L 3G1, Canada

**Keywords:** Nitrogen vacancy center in diamond, Acoustic wave, Asymmetry of double-dip, ODMR

## Abstract

We report acoustic control of spin resonance in nitrogen-vacancy (NV^–^) centers in diamond using low-frequency free-space sound waves. Acoustic excitation redistributes spin populations between the |+1⟩ and |−1⟩ states, producing an asymmetric, tunable double-dip ODMR spectrum without shifting resonance frequencies. This contrasts with conventional magnetic-field-induced splitting and reveals a previously unobserved mechanism for spin manipulation via strain-mediated acoustic coupling. Analytical modeling captures the essential features of the amplitude flipping and elucidates the underlying spin-strain interaction. Our findings establish a new degree of freedom for selective multimodal quantum sensing and open avenues for hybrid spin-mechanical quantum devices.

## Graphical abstract

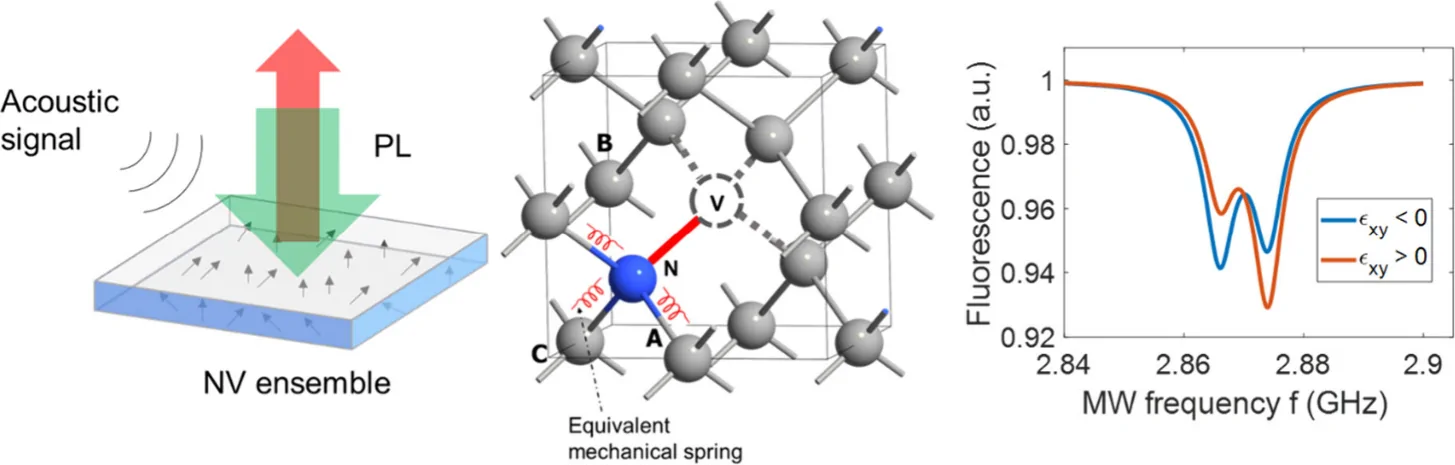

Negatively charged nitrogen vacancy (NV^–^) centers in diamond has been intensively researched as quantum sensors under various stimuli, such as magnetic field, mechanical strain, temperature, and electrostatic field.^1^ In majority of applications, the sensing function relies on ensemble of NV^–^ centers rather than single defects, which has advantage of operating at any temperature including room temperature, which makes it very attractive than other quantum sensing mechanisms such as Josephson junctions,^2^ superconductive quantum interference devices (SQUIDs),^3^ and optically pumped magnetometers (OPM)^4^ that require strict cryogenic conditions. Previously both in-depth theoretical and experimental research have been conducted for understanding the physics, optimizing the material growth, and characterizing the sensing effects. Notable research in theoretical development includes density functional theory (DFT) analysis of the diamond NV^–^ to uncover working principles and optimize the material growth.^5–8^ With regard to characterization, several techniques have been used to measure the quantum effects of the diamond NV^–^. It is often characterized with the continuous wave optically detected magnetic resonance (CW-ODMR) technique,^9^ with which the photoresponse resonant spectrum along the microwave frequency is recorded with potential shifts being subjected to external magnetic fields, known as Zeeman splitting. Pulsed ODMR was also used to eliminate the optical and microwave broadening.^10^ Other techniques exist for various purposes, for example, Ramsey measurement,^11^ spin relaxation, and spin echo measurement.^12,13^ The basic physics foundation of the Diamond NV^–^ has been well understood. It relies on the interaction of quantum defect states (ground state triplet (^3^A), excited state triplet (^3^E), and intermediate state singlet). In both the ^3^A and ^3^E triplet states, electron spins align with *m*_*s*_ = ±1, positioned above the antiparallel configuration at *m*_*s*_ = 0. The intermediate singlet state, by contrast, contains only the *m*_*s*_ = 0 spin. When an external magnetic field is applied parallel to the crystal’s *c*-axis along the defect axis, the degenerate *m*_*s*_ = ±1 levels split, a phenomenon known as the Zeeman effect,^14^ which forms the basis of magnetic field sensing function. In the absence of such a field, diamond NV centers exhibit zero-field splitting, producing an energy gap (∼2.87 GHz) between the ground spin states that forms the basis for quantum sensing applications.

There has been an interesting phenomenon that the ODMR spectrum of the diamond NV^–^ displays asymmetric |±1⟩ states, in terms of the dip amplitude. The effect has been reported attributing to local strain and electrical fields.^15–17^

Here, we demonstrate that low-frequency acoustic waves selectively redistribute population between |+1⟩ and |-1⟩ states, generating an asymmetric, tunable double-dip ODMR spectrum. Unlike magnetic-field-induced splitting, this effect controls transition strengths without shifting resonance frequencies, representing a previously unreported mechanism for acoustic-driven spin control. This finding establishes a novel degree of freedom for quantum manipulation, opening avenues for multimodal quantum sensing and hybrid spin-mechanical devices.

Our measurements are performed at room temperature in an ambient optical laboratory using an optically detected magnetic resonance (ODMR) setup based on a bulk diamond (single crystal diamond wafer: 3 mm × 3 mm × 0.5 mm) containing an ensemble of NV^–^ centers (concentration ∼300 ppb, Element Six, Ltd.). The experimental configuration (Figure 1a) consists of a 532 nm collimated laser for optical excitation, a 550 nm dichroic mirror combined with a 500 nm long-pass filter for fluorescence separation, and a microwave loop antenna with a resonant frequency of ∼2.9 GHz for spin manipulation. The laser module was from Thorlabs (CPS532) with a maximum power output of 4.5 mW. The microwave signal was generated with an evaluation board containing a voltage-controlled oscillator (VCO) and a synthesizer, amplified by the Mini-Circuit low-noise RF amplifier (ZRL-3500+). The output microwave power is around 16 dBm to the antenna. For optical excitation, the laser excitation is operated near or at optical saturation with maximum optical power. For microwave drive, the MW power is kept below saturation (considering microwave loss and impedance mismatch between passing through the antenna and interfaces). Photoluminescence from the NV^–^ centers is collected using a silicon free-space photodetector with switchable gain, interfaced with an Arduino-based acquisition system for signal readout and processing. A representative ODMR spectrum is shown in Figure 1b, exhibiting two resonance dips centered at approximately 2.866 and 2.874 GHz. The spectrum is well described by Lorentzian fitting, revealing a clear asymmetry in the relative amplitudes of the two dips. Such asymmetric ODMR responses from ensembles of NV^–^ centers in bulk diamond have been previously reported and attributed to local strain effects,^15^ while similar behavior in single NV^–^ centers has been explained by the combined influence of local electric fields and strain.^18^

**Figure 1:**
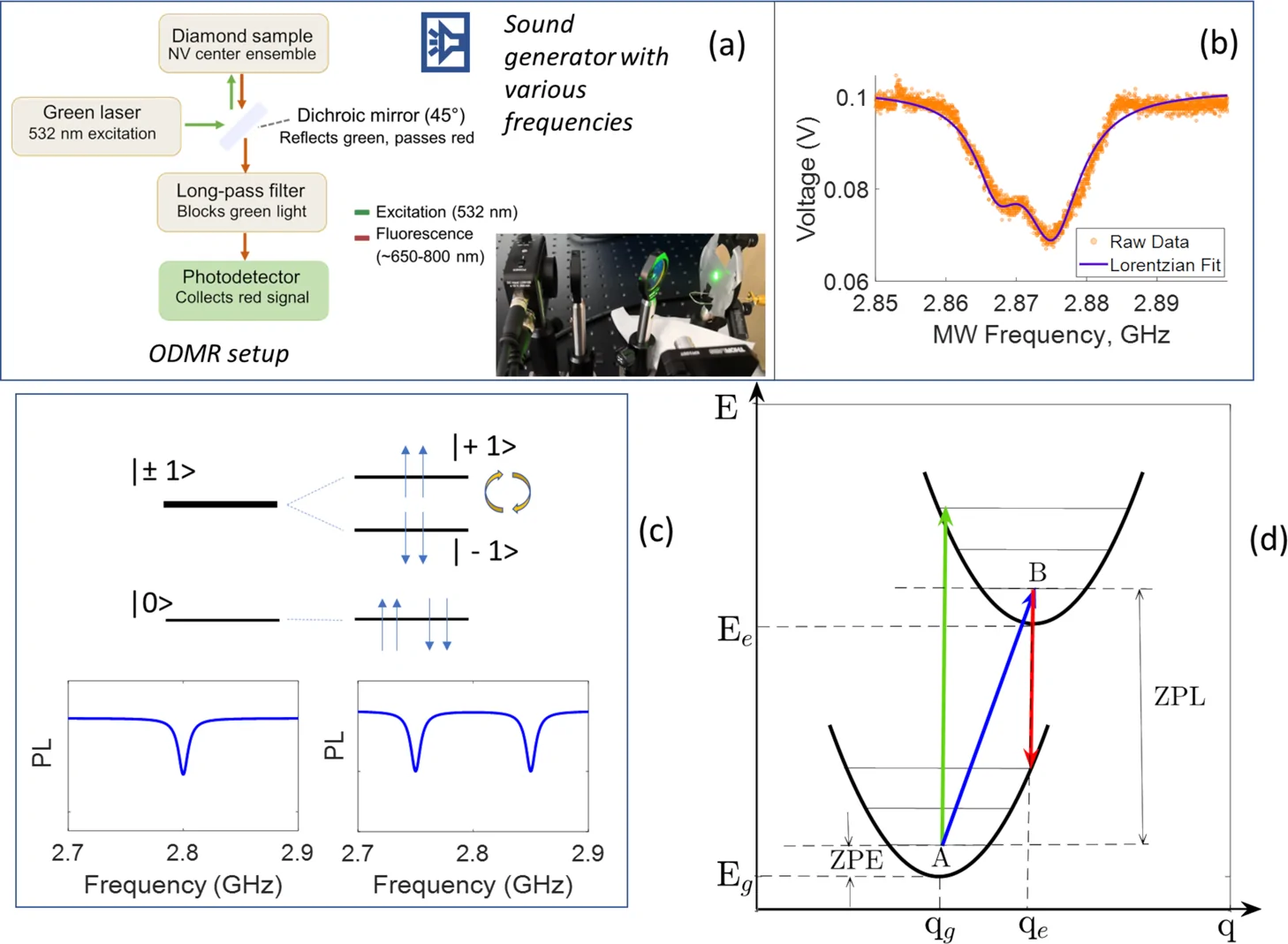
(a) Schematic of the experiment with a photo of experimental setup inserted. (b) One of measured ODMR spectra. (c) Ground-state triplet structure of the nitrogen-vacancy (NV^–^) center in diamond. In the absence of external perturbations, the spin sublevels |*m*_*s*_ = ±1⟩ are degenerate and separated from the |*m*_*s*_ = 0⟩ state by the zero-field splitting (∼2.87 GHz). Application of a magnetic field *B* along the NV axis lifts this degeneracy via the Zeeman effect, splitting the |+1⟩ and |−1⟩ states, which can be probed using optically detected magnetic resonance (ODMR). (d) Configuration coordinate diagram showing the energy (*E*) as a function of lattice configuration (*q*) for the NV center, illustrating the ground-state triplet, excited-state triplet, and intermediate singlet states involved in optical excitation and nonradiative relaxation pathways.

Fundamental of the diamond NV^–^ sensors is based on the paired defects that consists of a carbon vacancy and a nitrogen atom replacing a carbon atom. The energy level diagram is shown in Figure 1c. The paired defect center introduces a spin triplet ground state (|0⟩, |±1⟩), where the separation of the state |0⟩ and the |±1⟩ state is around 2.87 GHz due to zero-field splitting (ZFS). In the presence of external stimuli such as magnetic field and strain, the |±1⟩ state shift and split. With the presence of the magnetic field and without electrical field and strain, the general Hamiltonian is *H* = *hDS*_*z*_^2^ + *hγ*_e_ ***B*** · ***S***, where *h* is the Planck’s constant, *D* (2.87 GHz) is the ZFS, *γ*_e_ is the electron gyromagnetic ratio (∼2.8 MHz/G), and ***S*** = (*S*_*x*_,*S*_*y*_,*S*_*z*_) represents the spin – 1 Pauli matrices. Considering all effects including electric field and strain, the Hamiltonian becomes *H* = *hDS*_*z*_^2^ + *hγ*_e_ ***B*** · ***S*** + *H*_E_ + *H*_ε_, where *H*_E_ and *H*_ε_ are the spin-electric field and spin-strain Hamiltonians, respectively.^19^ It is shown schematically in Figure 1c that a single-dip ODMR spectrum reflects the degenerate |±1⟩ state, whereas a double-dip spectrum is for the splitting |±1⟩. It is schematically depicted that the flipping effect between |+1⟩ and |−1⟩ states under the acoustic excitations. Figure 1d shows the adiabatic potential energy surface of the ground state and excited states. The quasi-parabolic curves are energy representation of the potential well of a quantum harmonic resonator, in which the energy is quantized with discrete energy levels. The minima of the ground and excited states are denoted as *E*_g_ and *E*_e_, respectively, and *q*_g_ and *q*_e_ are correspondences in the *q*-axis. The energy ladders in both ground and excited states represent the phonon energies, and higher phonon energy ladders will be occupied with elevated temperatures. Zero-phonon energy (ZPE) is shown for the ground state. Energy transitions (vertical absorption, green arrow; vertical emission, red arrow) are due to absorption and emission of phonon energy. Transition A → B corresponds to the zero- phonon line (ZPL, blue arrow) in absorption. For the negatively charged NV^–^ centers in diamond, the ZPL is located at ∼637 nm.

Figure 2 presents the ODMR measurements under a series of acoustic signals with increasing frequencies and a fixed amplitude. From the experiment, the amplitudes of the two dips change, meaning that the acoustic waves are coupled into the crystal, making impact on the coupling strengths between microwave and quantum states, reflected by the change of florescence lightness. Acoustic waves at 1–2 kHz lead to the first dip at lower microwave frequency having smaller luminescence value than the second dip. This indicates that the photoluminescence at *f*_2_ is brighter than that of *f*_1_, implying that there is stronger microwave resonance at the |−1⟩ state than that at |+1⟩ state. For acoustic waves at 3–6 kHz, this has changed to the opposite, showing a clear transition from stronger microwave resonance at |−1⟩ state to |+1⟩ state. As the microwave frequency increases to 7 kHz and above, the second dip becomes brighter than the first, showing that the microwave resonance for the |+1⟩ state gets weaker. This flipping effect indicates there is coupling between the external acoustic waves and the microwave resonances of the ground triplet in the diamond NV^–^ ensemble.

**Figure 2:**
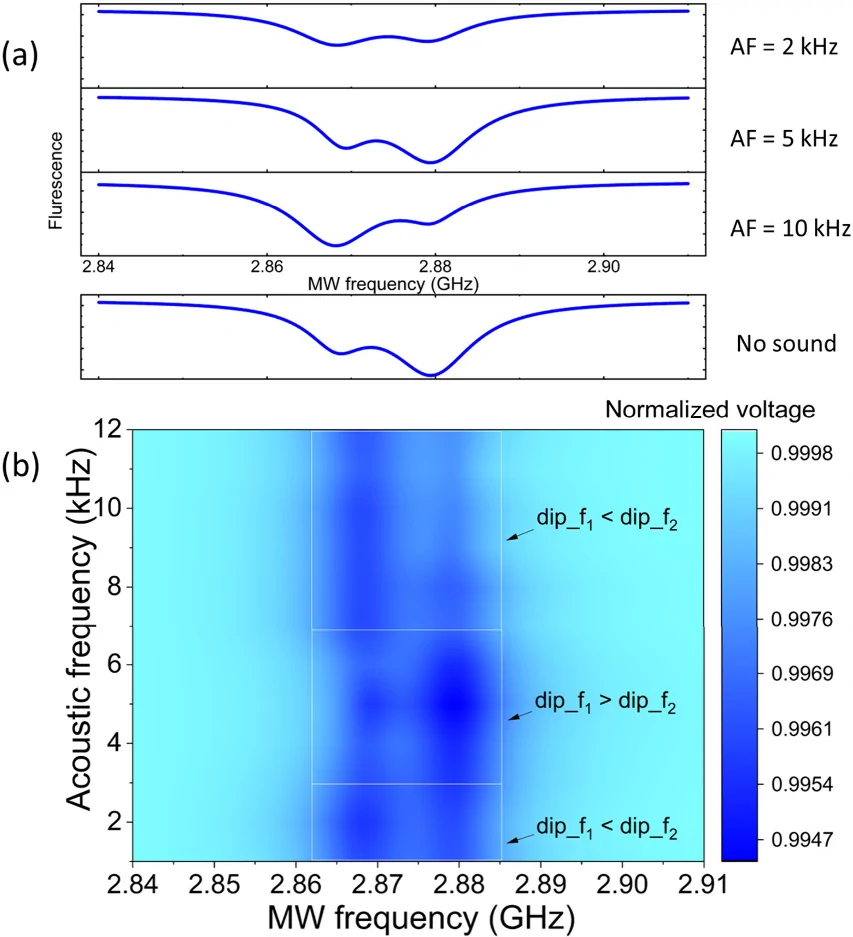
(a) Optically detected magnetic resonance (ODMR) spectra in the frequency range 2.84–2.91 GHz under various acoustic excitations. The evolution of the spectra reveals a pronounced modulation of the double-dip structure, including a clear amplitude “flipping” effect in which the relative depths of the two resonance dips are reversed as a function of acoustic frequency. This behavior indicates acoustic-wave-induced redistribution of the spin-state populations associated with the |0⟩ ↔ |±1⟩ transitions. (b) Three-dimensional representation of the ODMR spectra as a function of microwave frequency and applied acoustic excitation, illustrating the continuous evolution of the spectral line shape. The plot highlights the dynamic modulation of dip amplitudes and the emergence of asymmetry driven by the external acoustic field.

It is seen in Figure 3 that the minima of luminescence amplitudes (normalized) of the double-dip ODMR spectra switch over between acoustic driving signals in the ranges of 2–3 kHz and 6–7 kHz (Figure 3a). This can be represented by the strengths of the dips α_–_ and α_+_ from the Lorentzian fittings for the two dips in the ODMR spectra. α_–_ and α_+_ were extracted from the Lorentzian equation used to fit the ODMR measurements. At least four measurements have been recorded for one set of fixed experimental conditions to arrive at an averaged spectrum. The spectrum based on the Lorentzian equation with the width γ and frequency *f* is

1 $$L\left(f\right)=\frac{\gamma }{f^{2}+\gamma^{2}}$$

with the Lorentzian, the microwave spectrum is written for NV^–^ ensemble as

2 $$P\left(f\right)=1-A\left[\alpha_{-}L\left(f-f_{1}\right)+\alpha_{-}L\left(f-f_{2}\right)\right]$$

where *A* is the arbitrary scaling amplitude, *f*_*1*_ and *f*_*2*_ are frequencies of two dips in the spectra, with *f*_1_ being the lower frequency. In Figure 3b, α_–_ and α_+_ are tuned by the acoustic waves with different acoustic frequencies. The dip strength at higher frequency point α_+_ shows greater tunability in the range of 0.16–0.53, around 2.5 times wider, compared with the tuning range of 0.25–0.4 for α_–_. *A* was set to a fixed value when extracting α_–_ and α_+_.

**Figure 3:**
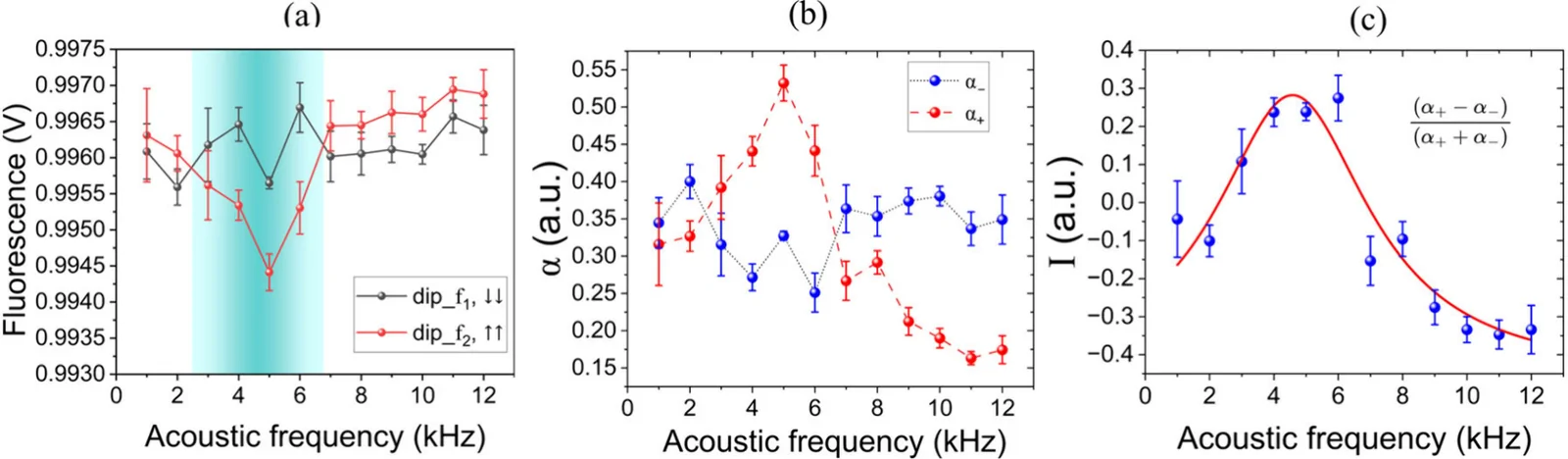
(a, b) Extracted amplitudes of the two resonance dips in the ODMR spectra as a function of applied acoustic frequency. The quantities dip_*f*1_ and dip_*f*2_ correspond to the transition amplitudes associated with the |0⟩ ↔ |−1⟩ and |0⟩ ↔ |+1⟩ spin states, respectively. The evolution of these amplitudes with acoustic excitation reveals a redistribution of spin populations and a clear modification of the ODMR line shape. (c) Extracted imbalance parameter *I*, defined to quantify the relative asymmetry between the two dips, highlighting the acoustic-frequency-dependent modulation of the ODMR spectrum. The variation of *I* demonstrates the emergence and reversal (“flipping”) of spectral asymmetry induced by acoustic waves.

The degree of asymmetry can be quantified with the imbalance *I*,^15^ which is defined as

3 $$I=\frac{\alpha_{+}-\alpha_{-}}{\alpha_{+}+\alpha_{-}}$$

From extracted α_–_ and α_+_, *I* was calculated with acoustic frequencies, shown in Figure 3c. The data was further fitted with the Lorentzian model,

4 $$I\left(f_{a}\right)=I_{0}+\frac{\int \left(I-I_{0}\right)\;df_{a}}{\pi }\cdot \frac{\Gamma }{4{\left(f_{a}-f_{ac}\right)}^{2}+\Gamma^{2}}$$

where *f*_*a*_ is the acoustic frequency, *f*_*ac*_ is the frequency at which the *I* is at the peak. The term ∫(*I* – *I*_0_) d*f*_*a*_ represents the integrated area under the curve. Γ is the half-width at half-maximum, *I*_0_ denotes the offset. From fitting the data, the key parameters in eq 4 are as follows: imbalance *I* has a resonance point at an acoustic frequency of 4.6 kHz, and the quality factor is ∼0.78. It was described in ref 15 that the asymmetric double-dip in ODMR is due to strain effect, and analytical model was also reported. In the single NV model, the degree of asymmetry *I*,

5 $$I=-\cos\left(2\phi_{\mathrm{MW}}+\phi_{\mathrm{str}}\right)$$

where ϕ_MW_ is the azimuthal angle between the microwave wave projected in the NV plane and *x*-axis of the NV center. According to ref 15, an approximated ϕ_MW_ is taken as π/2 for the case of microwave (MW) direction projected in *y*-axis in the NV plane. Therefore, the main contribution to the asymmetry is the effective strain “vector” in-plane angle, ϕ_str_ = $\tan^{-1}\left(\frac{M_{y}}{M_{x}}\right)$ , where *M*_*x*_ and *M*_*y*_ are strain amplitudes that are related to the strain components in the N–V coordinate ε_*xx*_, ε_*yy*_, ε_*zz*_, ε_*xy*_, ε_*xz*_, ε_*yz*_:

6 $$M_{x}=\frac{1}{2}\left[h_{16}\varepsilon_{xz}-\frac{1}{2}h_{15}\left(\varepsilon_{xx}-\varepsilon_{yy}\right)\right]$$

7 $$M_{y}=\frac{1}{2}\left(h_{16}\varepsilon_{yz}+h_{15}\varepsilon_{xy}\right)$$

where *h*_*ij*_ are defined spin-strain coupling strengths.^19^ For numerically calculating asymmetric ODMR, *h*_15_ = 5.7 ± 0.07; *h*_16_ = 19.66 ± 0.09, strain tensors eqs 6 and 7 can be taken as ε_*xx*_ = −ε_*yy*_ = 10^–4^; ε_*zz*_ = ε_*yz*_ = ε_*xz*_ = 0 while varying the shear strain ε_*xy*_.

Figure 4a depicts the acoustic effect on the diamond chip containing NV^–^ ensemble, and Figure 4b shows the atomic structure of a diamond crystal consisting of a single NV^–^ pair. Based on eqs 5–7, the imbalance of the double-dip is calculated and is shown in Figure 4c.

**Figure 4:**
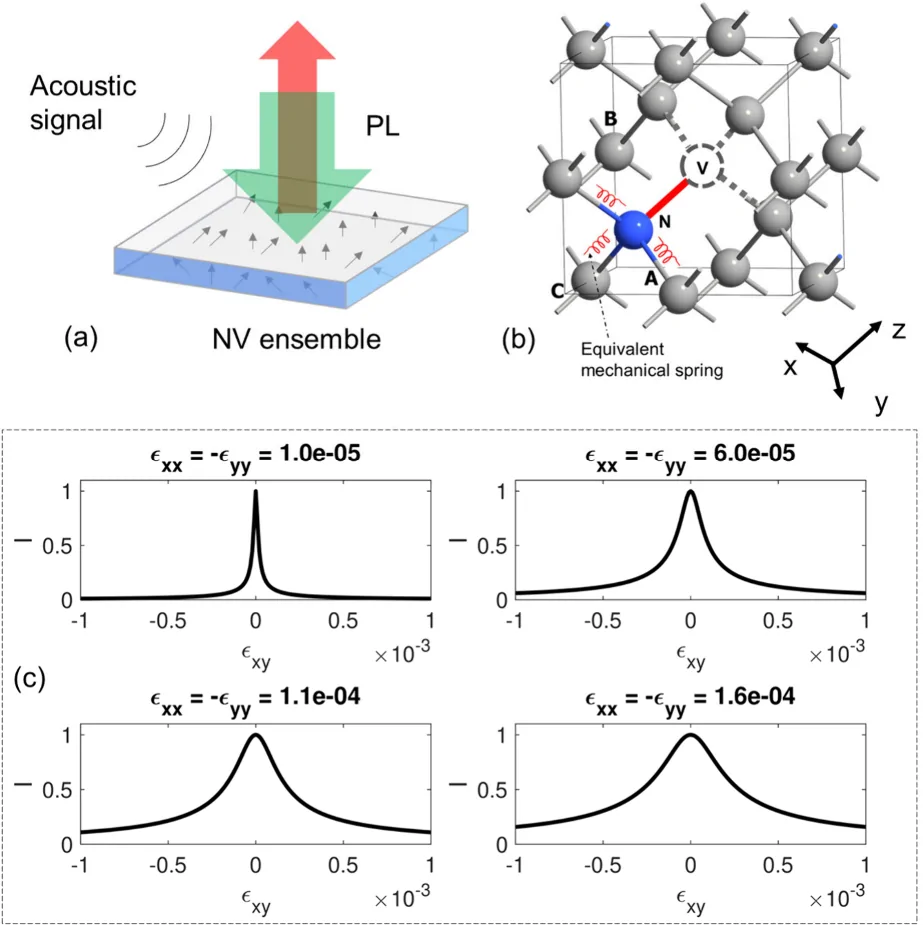
(a) Acoustic signal impacting the luminescence of the diamond NV ensemble; (b) NV [1 1 1] with *z*-axis aligning to the direction of NV^–^ pair, and *x*-axis is one of N–C–V plane. Carbon atom is shown in gray, nitrogen is in blue, and vacancy is in dashed line, red line connects N and V; equivalent mechanical spring for N–C bonds. (c) strain effect modeling on the double-dip imbalance.

The results indicate that the imbalance is sensitive not only to the shear strain in *x*–*y* plane, but also to the in-plane axial strains. The axial strains ε_*xx*_ and ε_*yy*_ affect the width (sharpness) of the imbalance curve. The imbalance *I* is at the largest when the additional shear strain ε_*xy*_ = 0, which is determined by the relative angle between MW and strain directions. Increasing shear strain rotates the strain vector away from *x* toward to *y* (MW direction), making the two transitions more balanced. Purely considering the strain effect, additional strain will cause three types of changes in ODMR: (i) shift of the central MW frequency; (ii) splitting of the dips (superpositions); and (iii) imbalance of the dips. Clearly, the free space acoustic waves within the low-frequency spectrum (1–12 kHz) cause the change of imbalanced double-dip shown in Figure 3, extracted from the experiment, unveiling that the acoustic signals only lead to coupling to the in-plane strains (*x*–*y* plane), no coupling to volumetric strain and shear strains in the *y*–*z* plane, since there is no shift of central ODMR frequency, and no effect on splitting. This is a special and unreported phenomenon where imbalance *I* changes but there is no frequency splitting and shift taking place, meaning the strain modifies the transition strengths without introducing off-diagonal mixing between |+1⟩ and |−1⟩. It means there are small shear or off-diagonal strains that modify the coupling strength, but not energies. In summary, based on existing spin-strain coupling theory, the strain conditions for this scenario are (i) ε_*xx*_ = −ε_*yy*_ ≠ 0, Δ*f*_0_ = 0 (no shift, Δ*f*_0_ = 0); (ii) $\sqrt{M_{x}^{2}+M_{y}^{2}}$ = constant (fixed splitting δ*f*); (iii) the strain angle ϕ_str_ varies. Hence, the prominent discovery from this work is that the diamond NV sensor has the capability to distinguish the magnetic field and acoustic signals from the ODMR measurements. Previously phonon dissipation in the crystal lattice was calculated as few kHz in diamond NV,^20^ setting the testing condition of the acoustic frequencies chosen in the experiment presented in this work. From an electronic point of view, NV spin center creates localized quantized energy levels with preserved spin properties that can be modulated by external magnetic field. Viewing it mechanically, the actual bonds between a nitrogen atom and surrounding carbon atoms can be regarded as elastic springs (red spiral springs in Figure 4b), the carbon vacancy adjacent to the nitrogen atom effectively removes the mechanical “spring”, which blocks/weakens the transmission of mechanical vibrations along the NV axis., i.e., phonon conduction is blocked (or significantly weakened) along the NV axis. Hence, although the free space acoustic waves impose global pressure to the diamond wafer, the phonon conduction along the NV axis is much smaller than other directions through the actual atomic bonds. Macroscopically, it reflects to a lower thermal conductivity for diamond wafer containing NV ensemble. Evidence of lower thermal conductivity in diamond chip with dense NV centers was reported previously.^21^ It is known that the defect centers are randomly positioned in the crystal, and for the reasons described above, the acoustic waves mainly induce mechanical strains in directions other than the NV axis, which explains that the acoustic waves make impact on the asymmetry of the double dip in ODMR spectra. The free space acoustic driven enables the theory of strain-spin coupling for a single NV center being applicable to the case of the ensemble of NV centers, as the virtual NV mechanical bond filters the phonon dissipation along the NV axis. Hence, simulations with the single NV model were used for amplitudes flipping effect of the ODMR spectrum and imbalance level *I*. The results in Figure 5 present the amplitude flipping of the double dips, and imbalance *I* against a range of shear strains. A small perturbation was introduced to ϕ_MW_, to induce the amplitude flipping effect. The curves in Figure 5 show greater similarity to the experiment. Figure 6 presents ODMR spectra recorded under continuous 5 kHz acoustic excitation at three measured acoustic levels: low, 86.6 dB­(C); medium, 92.0 dB­(C); and high, 95.5 dB­(C). The background sound pressure level was 59.2 dB­(C). In the following calculation, additional 1.3 dB is added to these values due to C-weighting measurement. The corresponding incident acoustic intensities for the above sound level conditions are 5.99 × 10^–4^, 2.08 × 10^–3^, and 4.65 × 10^–3^ W m^–2^, respectively. The acoustic levels were measured at the sample position using a Casella CEL-24X sound level meter operated with C-frequency weighting and slow time weighting. The acoustic source was directed toward the sample surface from approximately 20 cm. Under all three acoustic conditions, Dip 1, centered near 2.865 GHz, exhibited a larger absolute ODMR contrast than Dip 2, centered near 2.887 GHz. No reversal in the relative dip contrasts was observed as the acoustic amplitude was increased. The absolute contrasts of both resonances showed only a modest amplitude-dependent variation. It can be interpreted as follows: acoustic waves are received by the holding element (PET film), then transferred to and absorbed by the diamond chip. First, the acoustic pressure amplitude *P*_amp_ corresponding to given sound pressure level *L*_sp_ is predicted using the standard reference pressure *P*_0_ of 20 μPa, *P*_amp_ = $\sqrt{2}\cdot P_{0}\cdot 10^{L_{sp}/20}$ ≈ 1.96 Pa. The maximum bending moment at the center of the clamped PET fixture film is estimated as *M*_max_ = β_M_ *P*_amp_α^2^·*AmpFactor*, where β_M_ is the dimensionless plate-bending coefficient for a clamped circular plate (∼0.086), and α is the free (clamped) radius of the fixture plate (taken here as approximately 3 mm). The dynamic amplification factor for harmonic loading, *AmpFactor* = $1/\sqrt{{\left(1-r^{2}\right)}^{2}+{\left(r/Q\right)}^{2}}$ , with *r* = *f*/*f*_0_ the ratio of excitation frequency *f* to the resonant frequency *f*_0_ of the film, and *Q* the quality factor of the film (∼20). The excitation frequency was fixed at *f* = 5 kHz, matching the acoustic drive used in the experiment. The resonant frequency *f*_0_ of the clamped PET film was estimated from classical circular-plate vibration theory to be approximately 3.3 kHz. The bonded diamond chip modifies both the effective stiffness and modal mass of the fixture; therefore, the actual resonance frequency of the complete assembly may differ from the PET-only estimate and may be higher if the added stiffness dominates. With the predicted bending stiffness *S*_eff_ ≈ *E*·*h* ^3^/[12­(1 – ν^2^) ≈ 1.9 × 10^–5^ N m], with PET film’s Young’s modulus *E*_PET_ ≈ 3 GPa, Poisson’s ratio ν_PET_ ≈ 0.4, and thickness *h*_PET_ ≈ 40 μm), the strain transferred to diamond chip using a sound level of 95.5 dB­(C) can be approximated by ε_diamond_ = (*t*_diamond_)·(*M*_max_/*S*_eff_), where *t*_diamond_ is the thickness of diamond chip. Substituting relevant parameters into the above derivation procedure, the induced in-plane strain in the diamond is ∼3.17 × 10^–5^. Since axisymmetric plate bending produces no off-diagonal (shear) strain by symmetry, the shear strain in Figure 5 was not derived directly from the mechanical model but instead constrained by fitting the spin-strain simulation to the experimentally observed ODMR imbalance. Relative to the estimated axial bending strain (∼3.17 × 10^–5^), a shear-to-axial ratio η ≈ 0.1–0.3 (ε_*xy*_ ≈ 10^–6^) reproduces the observed behavior, indicating that shear strain is a modest (10%–30%) correction to the dominant axial component. This ratio is reasonable in magnitude with the experimentally measured imbalance *I* in Figure 3c (ranging roughly between 0 and 0.3), further supporting the plausibility of this shear-strain estimate. This magnitude also aligns with a small geometric or alignment asymmetry—such as imperfect circular clamping, nonuniform tape adhesion, misalignment between the tape’s bending axes, and the NV crystallographic frame, or slightly non-normal acoustic incidence—rather than an independently driven shear mechanism, supporting the picture of shear strain as a secondary, symmetry-breaking perturbation. It is also worth noting that direct free-space acoustic coupling, which is dominated by impedance mismatch at the air–diamond interface, produces much smaller strains, on the order of 10^–12^ to 10^–14^, as according to Hooke’s law, ε = σ/*E*_diamond_, the sound induced stress σ = *T*_p_·*P*_amp_, where *T*_p_ = 2*Z*_2_/(*Z*_1_ + *Z*_2_), with *Z*_1_ and *Z*_2_ are impedances of air and diamond, respectively (*T*_p_ is close to 2 as the diamond’s acoustic impedance is ∼10^5^ times that of air), and *E*_diamond_ is ∼1050 GPa.

**Figure 5:**
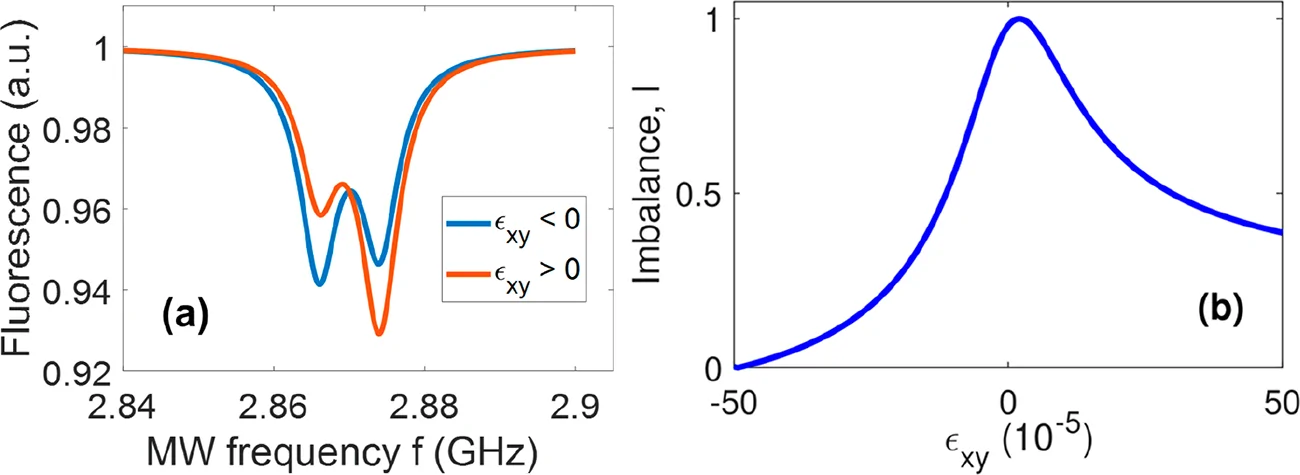
Numerical simulation based on a single NV spin–strain coupling model, incorporating a small perturbation (0.1) in the microwave phase ϕ_MW_ to reproduce the experimentally observed amplitude flipping between the two ODMR dips. (a) Simulated ODMR spectra under varying shear strain components, while the in-plane strain components are held constant at ±10^–5^. The results show that shear strain induces a redistribution of transition strengths between the |0⟩ ↔ |±1⟩ spin states, leading to a reversal of the relative dip amplitudes. (b) Extracted imbalance parameter *I* as a function of applied shear strain, quantifying the evolution of spectral asymmetry. The trend demonstrates a continuous tuning and eventual flipping of the ODMR dip imbalance driven by strain, consistent with the proposed acoustic–spin coupling mechanism.

**Figure 6:**
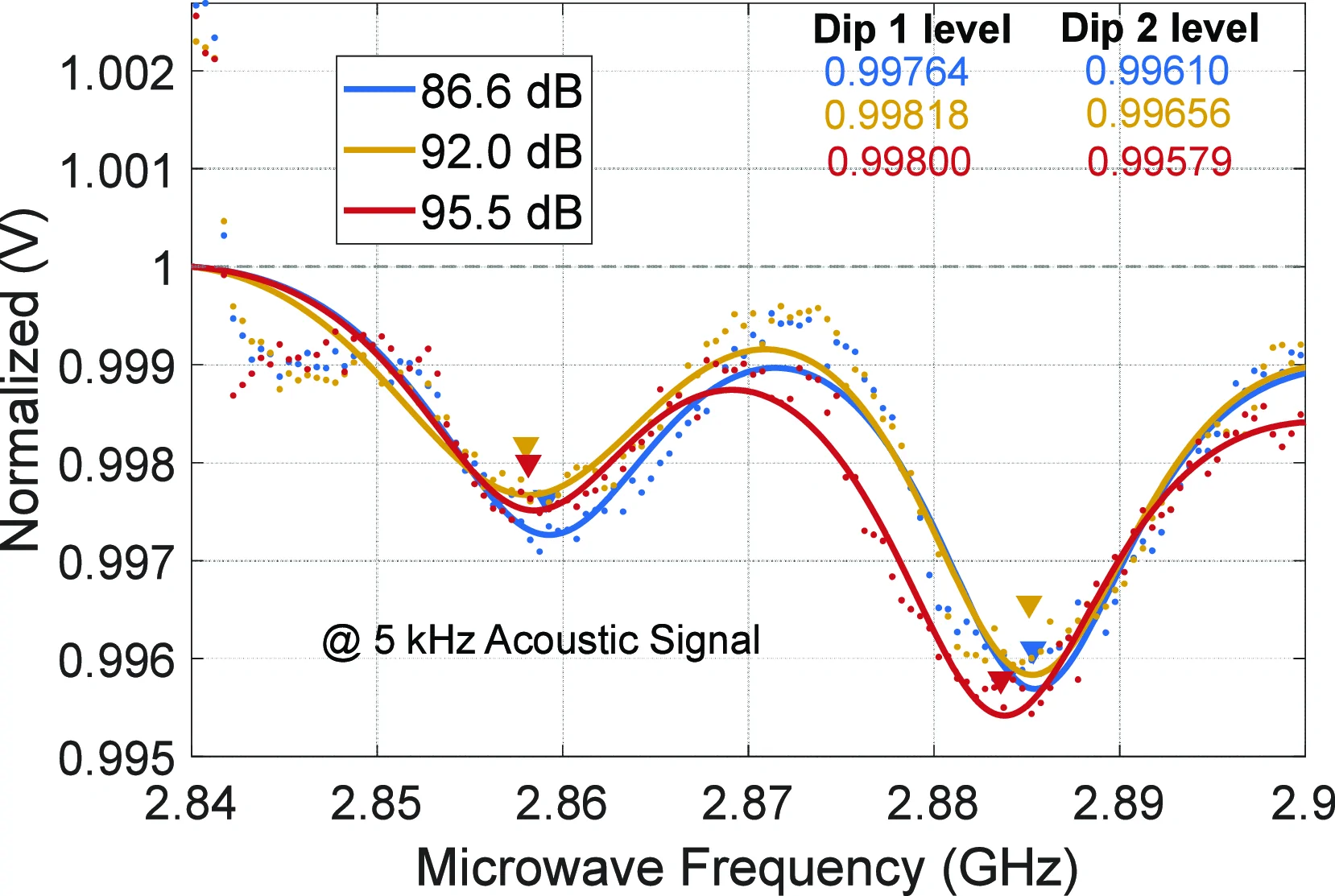
Normalized fluorescence as a function of microwave frequency for a 5 kHz acoustic drive at 86.6 dB (blue), 92.0 dB (orange), and 95.5 dB (red) amplitude. The background noise is measured to be 59.2 dB. Solid curves are Lorentzian fits to the raw data (dots); the dashed line indicates the maximum baseline (*V* = 1).

The observed frequency dependence should not be interpreted as an intrinsic resonance of the NV spin system, since the spin-strain Hamiltonian depends on the instantaneous local strain tensor rather than the acoustic drive frequency itself. Different mechanical modes generate different spatial distributions of tensile, compressive, and shear strain within the diamond and its mounting structure. Consequently, the local strain tensor experienced by a given NV ensemble changes with excitation frequency, leading to a frequency-dependent rotation of the effective in-plane strain vector entering the spin-strain Hamiltonian. Within our model, this changes the strain angle relative to the fixed microwave orientation and therefore modifies the imbalance *I* = −cos­(2ϕ_MW_ + ϕ_str_); a sufficient change in this relative orientation reverses the sign of *I*, accounting for the observed flipping of the ODMR dip amplitudes. The NV ensemble is comprised of centers aligned along the ⟨111⟩ crystallographic axis. As the diamond’s own N–C bond network acts as a mechanical filter that strongly attenuates phonon transmission along the NV axis, the acoustic-induced strain that couples to the spin states is dominated by the in-plane component normal to ⟨111⟩ for every NV center in the ensemble. This orientation-independent, axially filtered coupling geometry justifies extending the single-NV spin-strain Hamiltonian to model the ensemble-averaged ODMR response, since axial inhomogeneity, the main obstacle to single-defect-to-ensemble extrapolation, is suppressed by this mechanical filtering mechanism. Moreover, density functional theory (DFT) simulation has been conducted for a single spin center diamond NV system that is under mechanical strain (shown in the Supporting Information). Our intention here was not to model the complete ensemble quantitatively, but rather to identify the microscopic mechanism responsible for the experimentally observed ODMR amplitude modulation. The theoretical analysis therefore employs the well-established single-NV spin–strain Hamiltonian, which describes the fundamental interaction between local strain and the NV electronic spin. The theoretical model is therefore used as a microscopic effective model to demonstrate that strain-induced redistribution of the |+1⟩ and |−1⟩ spin populations naturally produce the observed amplitude asymmetry without requiring resonance-frequency shifts. Our objective is to reproduce the underlying physical mechanism and the qualitative behavior (including amplitude flipping and imbalance), rather than to perform a quantitative fit to the ensemble ODMR spectrum. Because acoustic-driven imbalance changes transition strength without shifting resonance frequency (unlike magnetic field, which shifts frequency), the two observables (frequency shift vs dip-imbalance) are mathematically independent readout channels. This means a single NV ensemble could simultaneously discriminate magnetic-field signals from acoustic/mechanical-strain signals in the same measurement, rather than needing separate sensor modalities, which is useful for multiplexed sensing in shared-environment applications (e.g., combined magneto-acoustic imaging). The large acoustic impedance mismatch between air and diamond means the NV ensemble only responds efficiently to acoustic energy under specific coupling conditions, effectively acting as a narrowband mechanical-to-optical transducer. This selectivity to frequency bands will be useful for underwater/biomedical acoustic sensing where cryogenic SQUID or OPM sensors are impractical at room temperature. Other potential applications include (a) acoustic-field sensing using NV centers without relying on resonance-frequency shifts; (b) hybrid spin–mechanical interfaces in which acoustic waves provide coherent control of spin populations; (c) multimodal quantum sensors capable of simultaneously detecting magnetic fields and mechanical vibrations through independent ODMR observables (frequency versus amplitude); (d) quantum microphones and mechanically addressable quantum transducers for low-frequency acoustic detection.

To summarize, in this work, acoustic signals are applied to the ODMR measurements of the diamond NV^–^ sensor. It is found that the free space acoustic wave is coupled to the diamond chip, causing the change of the asymmetry of the ODMR double dip. The asymmetry change is modulated by the acoustic frequency, and the acoustic frequency at which the acoustic-shear strain coupling reaches to the maximum is around 4.6 kHz, which is also accompanied by axial in-plane strains. There is no sign of the change in strain in *z*-axis (NV axis) in the presence of the acoustic signals, meaning the NV center significantly weakens phonon conduction. It is noted that the acoustic waves are dynamic signals, and the strain-spin coupling is mainly dominated by the phonon dissipation time in the crystal lattice. The results set the foundation for future multifunctional, selective sensing with a single quantum device.

## Supplementary Material



## Notes

Density functional theory (DFT) simulation of a single spin system on the projected density of states under various strain conditions, strain effect on NV defect levels, and the exchange splitting effect (PDF)
