# Supplementary material for: Acoustic Control of Spin States in Diamond Nitrogen-Vacancy Centres
Source: Nano Lett. 2026 Aug 12;26(33):11079–85. doi: 10.1021/acs.nanolett.6c02496 (PMC13523745; doi:10.1021/acs.nanolett.6c02496)
Supplement: Supplementary file 1 [file nl6c02496_si_001.pdf]

Supporting materials

Acoustic Control of Spin States in Diamond Nitrogen-Vacancy Centres

Emirhan Kutsal,[1] Amir Farokh Payam,[2] Eihab Abdel-Rahman,[3,4] Mustafa Yavuz,[3,5] Lijie Li[1]

[1]Department of Electronic and Electrical Engineering, Swansea University, Swansea, SA1 8EN, UK

[2]Nanotechnology and Integrated Bioengineering Centre, School of Engineering, Ulster University, Belfast, BT15 1AP, UK

[3]Waterloo Institute for Nanotechnology (WIN), University of Waterloo, Canada,

[4]Department of Systems Design Engineering University of Waterloo, Canada,

[5]Department of Mechanical and Mechatronics Engineering University of Waterloo, Canada.

Computational Details

All density functional theory (DFT) calculations were performed using the QuantumATK software package. The model system consisted of a 63-atom face-centred cubic (FCC) diamond supercell with composition $C_{62}N$, containing one nitrogen-vacancy (NV^-) defect pair. The atomic structure is shown in Figure S1. The supercell was constructed by substituting one carbon atom with nitrogen and removing an adjacent carbon atom to create the vacancy, with the N–V axis oriented along the crystallographic direction. Electronic structure calculations employed the HSE06 hybrid screened exchange-correlation functional, which accurately reproduces the localised defect states and band gap of diamond that standard semi-local functionals underestimate. Norm-conserving pseudopotentials from the PseudoDojo library were used to describe the core electrons, and valence electrons were expanded in a high-quality linear combination of atomic orbitals (LCAO) basis set as implemented in QuantumATK. The real-space mesh was defined by an energy cut-off of 75 Hartree, and Brillouin zone sampling was performed on a 2×2×2 Monkhorst–Pack k-point mesh, which is appropriate for this light-element supercell given the relatively weak dispersion of the localised NV defect states. A Fermi–Dirac electronic temperature broadening of 10 K was applied to aid convergence while effectively preserving the ground-state occupations.

The NV centre was treated as negatively charged (NV^-), consistent with the experimentally relevant spin-1 ground state. Spin-polarised calculations were initialised with an asymmetric spin configuration: a spin of +1 assigned to the nitrogen atom and a spin of −1 assigned to each of the three

carbon atoms neighbouring the vacancy, reflecting the antiparallel ferrimagnetic spin structure of the NV^- triplet ground state ($^3A_2$).

The projected density of states (PDOS) was computed for the nitrogen atom (site 24) and the three nearest-neighbour carbon atoms surrounding the vacancy (sites 14, 41, and 59), decomposed into spin-up and spin-down channels (Shown in Figure S2). This site-resolved projection allows direct identification of the NV defect levels within the bulk diamond band gap (~5.4 eV) and quantification of the local spin polarisation on each atomic site.

To investigate the effect of mechanical strain on the electronic and spin structure, two additional strained configurations were considered. First, a uniaxial tensile strain of 1% was applied along the NV axis (direction), elongating the N–C bond and directly modifying the axial component of the strain tensor. Second, a biaxial tensile strain of 1% was applied in the plane perpendicular to the NV axis, breaking the in-plane C_{3v} symmetry of the defect. For each strained geometry, the atomic positions were fully relaxed under the applied strain boundary conditions prior to recalculating the PDOS, ensuring the electronic structure reflects the equilibrium strained configuration. All three configurations (unstrained, axially strained, and transversely strained) were treated with identical functional, pseudopotential, basis set, k-point, and broadening settings to allow direct, unbiased comparison of strain-induced changes in the defect level positions, spin splitting, and local magnetic moments.

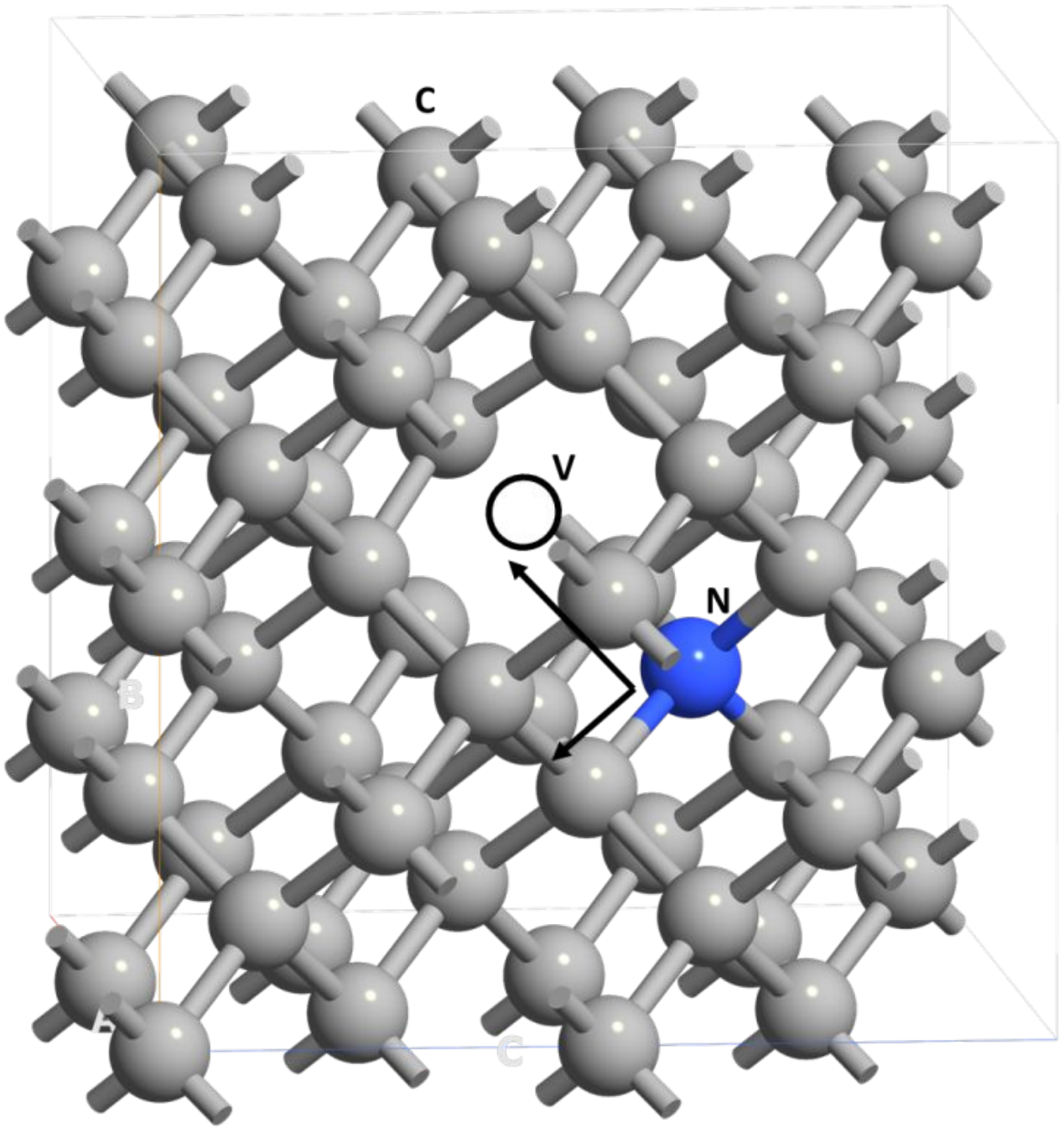

Figure S1, atomic structure of a NV centre in diamond.

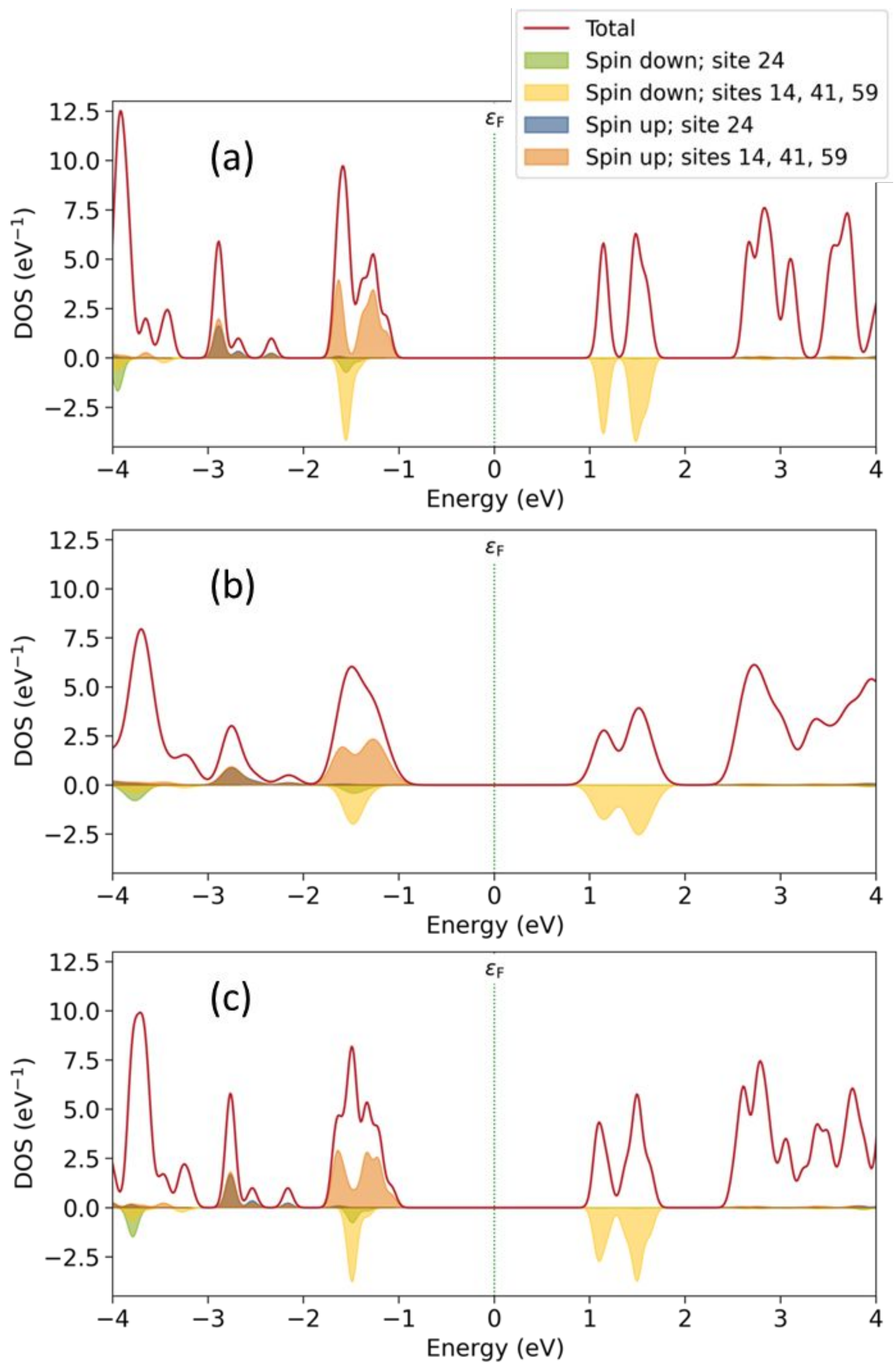

Figure S2, (a) project density of states for diamond NV- without strain; (b) with 1% tensile strain along the N-V axis; (c) with 1% tensile strain along the axis vertical to the N-V axis. Site 24 is the N atom, sites 14, 41, and 59 are 3 C vacancy neighbours.

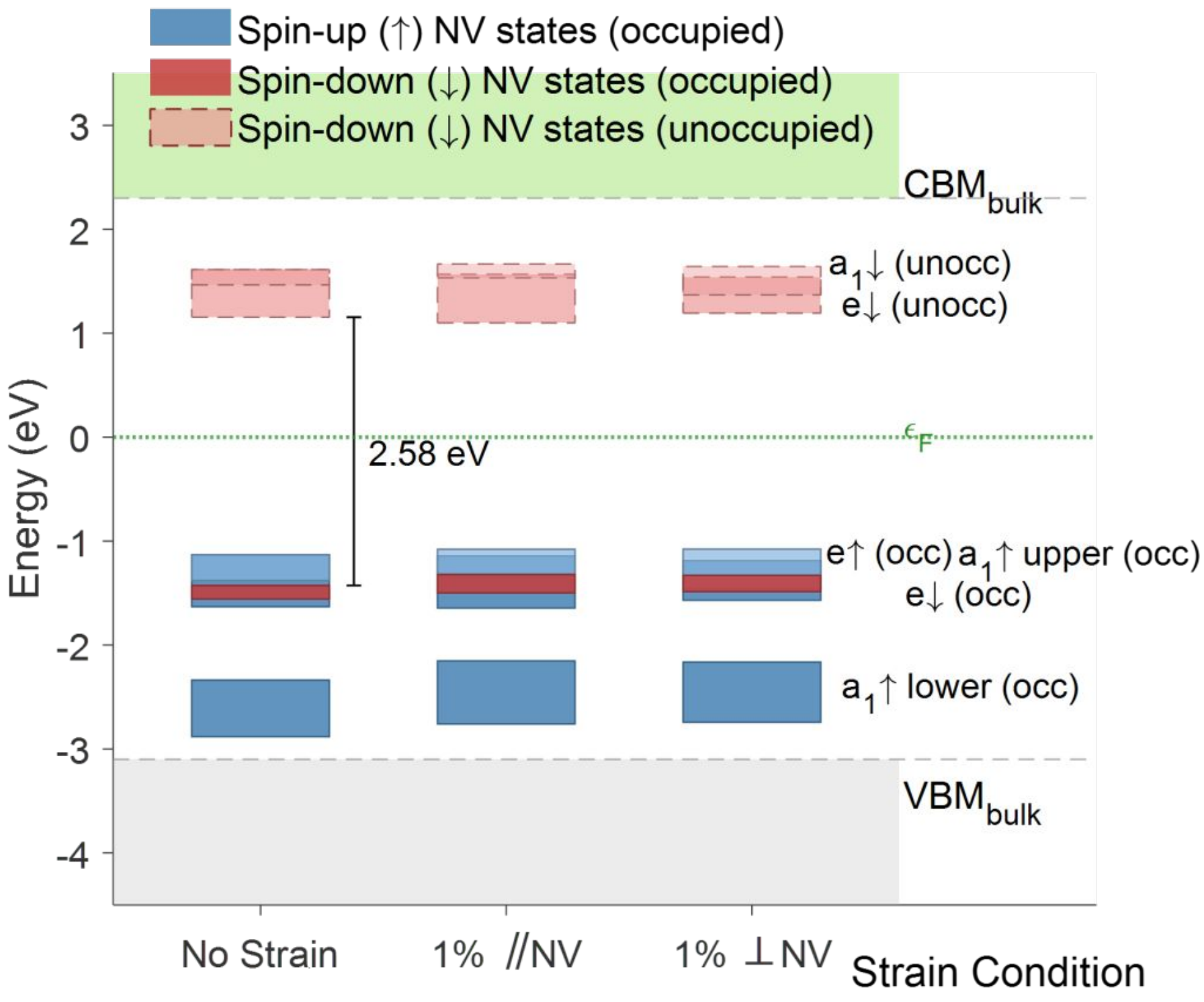

Figure S3, NV^- Defect State Energy Levels Inside Diamond Band Gap.
Bulk gap: VBM = -3.1 eV, CBM = +2.3 eV (gap ~ 5.4 eV).

The calculated bandstructure reveals six spin-resolved defect bands floating inside the bulk diamond gap, derived from the a1 and e symmetry orbitals of the NV^- centre in C_{3V}. Shown in Figure S3, the Fermi level lies within the NV spin gap — between the occupied ↓e state at ≈ −1.43 eV and the unoccupied ↓e state at ≈ +1.16 eV. This is the signature of the NV^- triplet ground state $^3A_2$: two unpaired electrons in degenerate e orbitals, with one filled (↑ channel) and the corresponding ↓ orbital left empty above ε_F. This 2.58 eV gap is the optical gap of the NV centre's spin manifold, directly related to the zero-phonon line (ZPL). The experimentally observed NV ZPL is ~1.945 eV (637 nm), with DFT hybrid calculations known to slightly overestimate this due to incomplete treatment of electron correlation in the gap.

Strain Effect on NV Defect Levels:

Shown in Figure S4a, axial strain (∥ NV) shifts the occupied ↓e top upward by +0.105 eV (toward ε_F) and shifts the unoccupied ↓e bottom downward by −0.055 eV (also toward ε_F). Both effects close the NV spin gap by a net −0.160 eV. Simultaneously, the ↑e top rises by +0.053 eV, increasing the spin-up/spin-down exchange splitting slightly. The a_1(lower) band top rises by +0.184 eV, indicating that the N–C bonding state along the NV axis is significantly destabilised by axial elongation. Physically, the N lone-pair couples more strongly to the vacancy when the bond is stretched.

Transverse strain (⊥ NV) has a distinctly different signature: the nominally degenerate ↑e doublet (bands 126↑ and 127↑) splits by 0.111 eV at the band top (−1.188 vs −1.077 eV), compared to zero splitting in the no-strain case and only 0.065 eV under axial strain. This e-orbital splitting is the Jahn-Teller–like lifting of the mₛ = ±1 degeneracy caused by in-plane symmetry breaking. It encodes the off-axis strain coupling parameter E in the NV spin Hamiltonian $\hat{\mathcal{H}}_{ZFS} = D\left(S_z^2 - \frac{S^2}{3}\right) + E\left(S_x^2 - S_y^2\right)$, where $\hat{\mathcal{H}}_{ZFS}$ is the zero field splitting, D is the axial ZFS parameter, S^2 is the total spin-squared operator. The unoccupied ↓e doublet (bands 126↓ and 127↓) also splits from a single band under no strain into two resolvable bands under transverse strain (separation: +0.176 eV at centre of mass), confirming the symmetry reduction from C_{3v} → Cs.

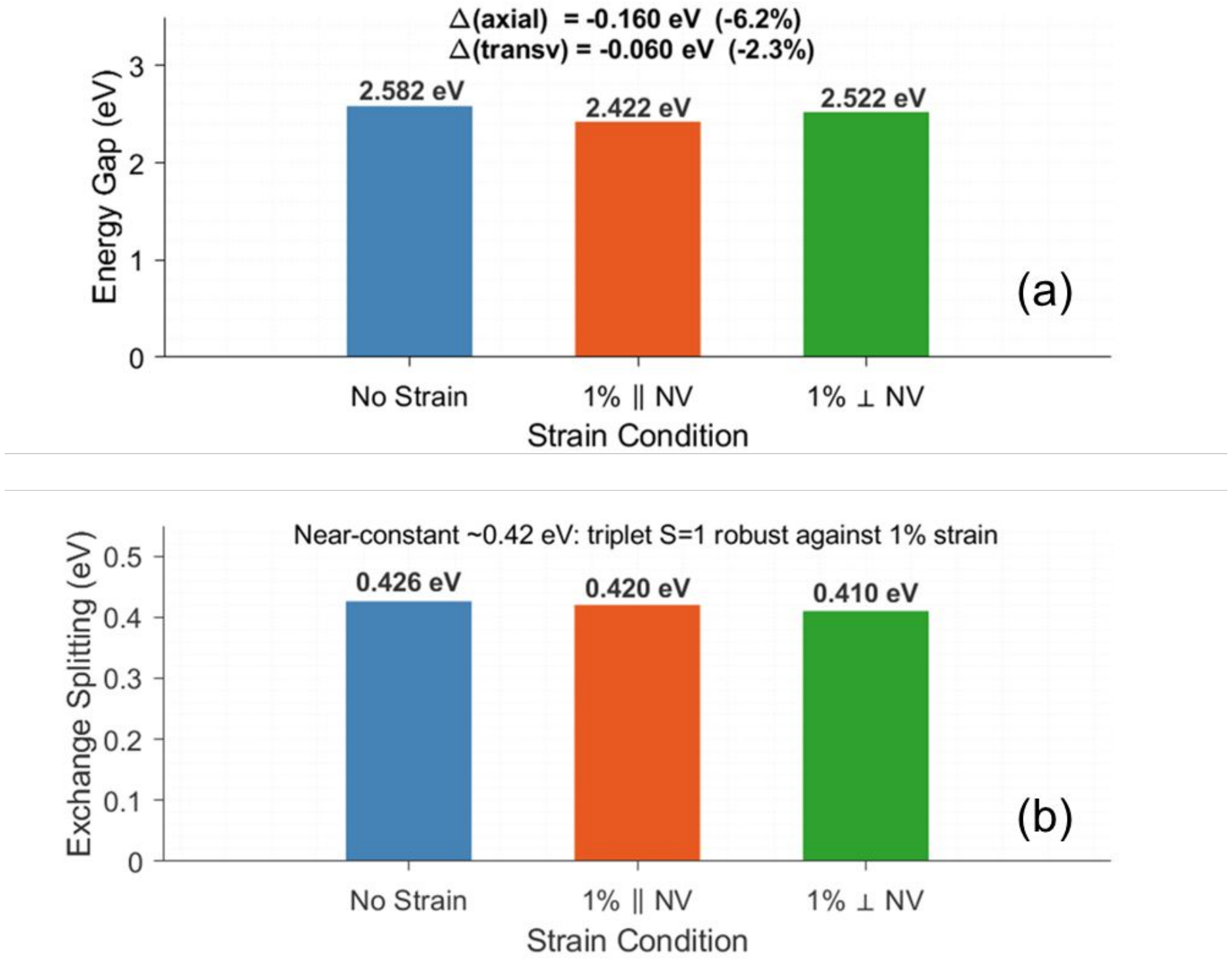

Figure S4, (a) NV Centre Spin Gap: Occupied → Unoccupied ↓ State. Gap between highest occupied e↓ and lowest empty e↓ band; (b) NV Centre Spin-Up/Down exchange splitting, top of occupied e↑ minus bottom of occupied e↓.

Exchange Splitting:

Shown in Figure S4b, the energy interval from the top of occupied ↑e (band 127↑) to the bottom of occupied ↓e (band 125↓) is strain-insensitive: The exchange splitting changes by less than 16 meV across all strain conditions, meaning the triplet spin state (S = 1) is topologically protected against 1% mechanical strain. Both spin-up and spin-down occupied e orbitals shift upward together under strain (rigid shift), preserving the inter-spin separation. This is consistent with the NV centre's established robustness as a qubit against decoherence from local strain — strain modifies the D and E ZFS

parameters (through the gap and e-splitting changes above) without destroying the spin-triplet character itself.

The 0.111 eV e-orbital doublet splitting under 1% transverse strain directly generates a non-zero E parameter. This is the direct physical origin of ODMR asymmetry: the two dips at $f_{\pm} = D \pm E$ separate, with one dip shifting up and the other shifting down from D.
